# Antimicrobial stewardship measures in cardiac surgery and its impact on surgical site infections

**DOI:** 10.1186/s13019-021-01693-7

**Published:** 2021-10-20

**Authors:** Güzin Surat, Dominik Bernsen, Christoph Schimmer

**Affiliations:** 1 Unit for Infection Control and Antimicrobial Stewardship, University Hospital of Würzburg, Würzburg, Germany; 2 Julius-Maximilians-University Würzburg, Würzburg, Germany; 3 Department of Cardiothoracic and Thoracic Vascular Surgery, University Hospital of Würzburg, Würzburg, Germany

**Keywords:** Antimicrobial stewardship, Surgical antimicrobial prophylaxis, Cardiac surgery, Surgical site infections

## Abstract

**Objective:**

The goal of this study was to monitor the compliance and impact on a protocol change of surgical antimicrobial prophylaxis in cardiac surgery favouring cefazolin instead cefuroxime, initiated by the hospital’s antimicrobial stewardship team.

**Methods:**

This quality improvement study was performed in a tertiary care hospital in collaboration with the department of cardiothoracic surgery and the hospitals antimicrobial stewardship team following a revision of the standard for surgical antimicrobial prophylaxis including 1029 patients who underwent cardiac surgery. 582 patients receiving cefuroxime and 447 patients receiving cefazolin respectively were compared without altering any other preventative perioperative measures including its postoperative duration of less than 24 h. Adherence and surgical site infections were compiled and analysed.

**Results:**

A complete adherence was achieved. Overall surgical site infections occurred in 37 (3.6%) of the cases, 20 (3.4%) in cefuroxime patients and 17 (3.8%) in cefazolin patients (p value = 0.754). No statistically significant differences could be found in any of the primary endpoints, but there was a trend towards less deep sternal wound infections in the cefazolin group.

**Conclusions:**

The study supports the role of antimicrobial stewardship in cardiac surgery and mirrors the success of a multidisciplinary team aiming to minimize adverse events by optimizing antibiotic use.

**Supplementary Information:**

The online version contains supplementary material available at 10.1186/s13019-021-01693-7.

## Introduction

Antibiotic prophylaxis in open-heart surgery has been an established procedure for decades. Regarding the invasiveness and mortality risks of postoperative deep sternal wound infections (DSWI) optimal antibiotic prophylaxis is crucial. Yet the recommended antibiotic regimen has changed many times over the decades and still varies with each center. The main principles of perioperative antimicrobial prophylaxis (PAP) include the timing, dosage and duration as well as the choice of the given agent. The most recommended agents for cardiac procedures belong to the class of cephalosporins [1]. The latest practice guidelines from the society of thoracic surgeons state that a first generation cephalosporin (mostly cefazolin) should be the primary choice for the PAP, suggesting a postoperative duration of 24–48 h [2, 3]. The majority of recommendations [1, 4, 5] regarding surgical antimicrobial prophylaxis (SAP) for cardiothoracic operations is based on studies and findings from the 1990s showing that the first-generation cephalosporin cefazolin is as effective as second-generation cephalosporins like cefamandole or cefuroxime for the prevention of surgical site infections (SSI) [6]. The university hospital of Würzburg (UKW) in Bavaria/Germany, launched an antimicrobial stewardship program (ASP) in July 2015 complying with the German Act on Prevention and Control of Infectious Diseases (Infektionsschutzgesetz §23) as response to the medical crisis due to increasing antimicrobial resistance [7]. ASPs are developed with the goal to improve the quality of antimicrobial use and to sustain the efficacy of antimicrobial drugs in the prevention and treatment of infections [8]. In accordance to national and international guidelines core elements of antimicrobial stewardship were implemented and in its wake antibiotic ward rounds and antimicrobial prescribing guidelines introduced [8, 9]. The existing in-hospital guideline on SAP was updated and amended in May 2017 affecting a dozen of surgical departments, favoring cefazolin as the new routine agent instead of the thitherto used cefuroxime. The main objective of this trial was to see through the new introduction of ASP in the UKW and to monitor its impact on compliance, SSI and risk factors. The duration with solely one post-operative dose was not touched with the change aiming to confirm the efficacy of a shortened SAP duration in adult cardiac surgery. The study reflects a multidisciplinary approach on improving the quality of antimicrobial use and was conducted in collaboration with the department of cardiothoracic surgery and the antimicrobial stewardship team (AMS) of the UKW.

## Methods

### Study design

This retrospective analysis involved 1029 inpatients who underwent coronary artery bypass grafting and/or valve surgery via median sternotomy admitted between July 30th, 2016 and December 27th, 2017 at the department of cardiothoracic surgery of the UKW. Patients who had preoperatively ongoing thoracic infections, were diagnosed with endocarditis or osteitis in two or more locations, patients who underwent heart transplantation or pregnant and breastfeeding women were excluded.

In line with the hospital new guideline on the use of SAP launched in May 2017, every patient received cefazolin for PAP, instead the previously used cefuroxime. Any prolongation of surgery (> 2 half-lives of the prophylactic agent) warrants additional intraoperative administration; meaning all patients in both groups received a further intraoperative dose. We compared 582 patients (group 1) with cefuroxime to 447 patients (group 2) with cefazolin. There was no difference concerning the pre-incision timing of administration (within 30 min before surgical incision), yet the duration was restricted in both groups to one dose 4 h after the last intraoperatively given dose. The dose for cefazolin with 2 g and cefuroxime with 1.5 g remained through out the same. The digital Meticillin-resistant *Staphylococcus aureus* (MRSA) scoring system used in the pre-assessment period before surgery did not identify any patients at elevated risks for MRSA colonization [10].

### Data collection

Data collection was performed from the patient’s files. All medical records were processed and analysed anonymously. This is a quality improvement study, so an ethics votum was not needed. All methods were carried out in accordance with relevant guidelines and regulations. The data collection involved patient’s preoperative status, the intraoperative course and the postoperative progress. The research followed a standardized survey sheet for every patient.

### Definitions

All patients were checked on a daily basis, especially on the progress of wound healing or any other signs of infections. Additionally, to the physical assessments full blood panels were performed. The applied end points of this study were carried out in compliance to the CDC-criteria [11]. The primary endpoints in this study were defined as overall SSI, including superficial surgical wound infections (SSWI), DSWI and infections of the vein harvesting site.

### Statistical analysis

The statistical analysis was performed using SPSS. A p value of ≤ 0.05 was defined as statistically significant. A descriptive overview over both groups was given and then analysed using the Mann–Whitney U Test for continuous variables and chi-squared test for categorical variables. This was done in order to determine statistical differences between the two groups. The endpoints were analysed using Chi-Square test and Mann–Whitney U test according to the scale type. Sub-group analyses were performed using the same tests for univariate analysis. A multiple logistic regression analysis was conducted subsequently to test and confirm the independency of the risk factors.

## Results

1029 patients could be included in the study. 582 (56.6%) patients received cefuroxime and 447 (43.4%) cefazolin as the prophylactic antibiotic. All patients were screened for preoperative characteristics (Table 1), comorbidities, intraoperative data and postoperative variables and treatments (Additional file 1: Tables S1–S4). Some significant statistical differences between the two patient groups were seen in the collected preoperative date as for creatinine and leukocyte levels. Analyzing the postoperative variables, additional differences regarding the intubation period, the drainage flow rate and the number of postoperative delirium could be identified. From the clinical point of view these differences can be interpreted as non-significant. Statistical tests indicate a significance because of the large study population even when the study groups practically show little to no difference (Additional file 1: Tables S1–S3).

**Table 1:** Preoperative patient data

	Total	Cefuroxime	Cefazolin	p
Patient number (%)	1029 (100)	582 (56.6)	447 (43.4)	
Sex (%)				
m	782 (76.0)	435 (74.7)	347 (77.6)	0.283
f	247 (24.0)	147 (25.3)	100 (22.4)	
Age (years)	68.2 ± 9.86	68.0 ± 10.11	68.48 ± 9.51	0.640
BMI (kg/m^2^)	28.72 ± 4.73	28.90 ± 4.68	28.48 ± 4.80	0.162
Normal weight	205 (21.3)	113 (20.7)	92 (22.0)	
Overweight	419 (43.5)	230 (42.2)	189 (45.1)	
BMI Grad (%) Class I Obesity	247 (25.6)	149 (27.3)	98 (23.4)	0.673
Class II Obesity	72 (7.5)	40 (7.3)	32 (7.6)	
Class III Obesity	21 (2.2)	13 (2.4)	8 (1.9)	
Creatinine (mg/dL)	1.32 ± 3.10	1.35 ± 4.02	1.28 ± 1.07	0.037
MDRD	72.0 ± 25.56	72.9 ± 24.86	70.9 ± 26.43	0.092
Leucocytes (1/nL)	8.09 ± 3.90	7.93 ± 4.51	8.30 ± 2.90	0.009
CRP (mg/L)	0.81 ± 2.22	0.73 ± 1.73	0.92 ± 2.73	0.578
HbA1c (%)	6.00 ± 1.00	6.01 ± 0.99	5.98 ± 1.01	0.296

Regarding the endpoints (Table 2) of this study no statistically significant differences between both study groups could be found. Overall 37 (3.6%) patients suffered some form of SSI, including DSWI in 18 patients (1.7%), and 17 patients (1.7%) had documented infections of the vein harvesting site. 20 (3.4%) total SSI occurred in the cefuroxime group compared to 17 (3.8%) in the cefazolin group (p = 0.754). DSWI could be diagnosed in 12 (2.1%) of the cefuroxime patients and in 6 patients (1.3%) from the cefazolin group (p = 0.383). SSWI are not listed. The overall mortality was 3.9% with 3.3% in the cefuroxime group an 4.7% in the cefazolin group (p = 0.238).

**Table 2:** Primary and secondary endpoints of the study

Endpoints of the study	Total	Cefuroxime	Cefazolin	p
SSI total (%)	37 (3.6)	20 (3.4)	17 (3.8)	0.754
DSWI (%)	18 (1.7)	12 (2.1)	6 (1.3)	0.383
Vein harvesting site (%)	17 (1.7)	7 (1.2)	10 (2.2)	0.197
Urinary tract infections (%)	40 (4.0)	26 (4.6)	14 (3.3)	0.306
Pneumonia/trachebronchitis (%)	70 (7.0)	41 (7.1)	29 (6.7)	0.818
Sepsis (%)	11 (1.1)	8 (1.4)	3 (0.7)	0.294
Death (%)	40 (3.9)	19 (3.3)	21 (4.7)	0.238

Furthermore we were able to determine risk factors within the entire study population for developing SSI in DSWI (Table 3). Univariate analyses showed significant risk factors were CRP ≥ 1 mg/d (p = 0.005), peripheral arterial disease (p = 0.009), renal insufficiency (p = 0.023), operating time (p = 0.000), revision operation (p = 0.000), myocardial infarction within half a year before surgery (p = 0.027), transfusions ≥ 5 (p = 0.000) and the duration of intubation (p = 0.031).

**Table 3:** Univariate analysis of patient risk groups

Infections	SSI	p	DSWI	p
Age (% within subgroup)				
≥ 80a	3 (2.9)	0.668	2 (1.9)	0.898
< 80a	34 (3.7)		16 (1.7)	
Sex (%)				
M	25 (3,2)	0.221	12 (1.5)	0.350
F	12 (4.9)		6 (2.4)	
BMI (%)				
≥ 30 kg/m^2^	16 (4.7)	0.179	9 (2.6)	0.123
< 30 kg/m^2^	21 (3.0)		9 (1.3)	
CRP (%)				
≥ 1 mg/dL	12 (7.4)	0.005	6 (3.7)	0.040
< 1 mg/dL	25 (2.9)		12 (1.4)	
COPD (%)				
Yes	5 (5.3)	0.348	3 (3,2)	0.264
No	32 (3.4)		15 (1.5)	
Diabetes mellitus (%)				
Yes	15 (4.6)	0.227	7 (2.2)	0.495
No	22 (3.1)		11 (1.6)	
Nicotine abuse (%)				
Yes	15 (4.3)	0.357	6 (1.7)	0.986
No	22 (3.2)		12 (1.8)	
Myocardial infarction within the past half year (%)				
Yes	12 (5.1)	0.152	8 (3.4)	0.027
No	25 (3.1)		10 (1.3)	
Ejection fraction < 30% (%)				
Yes	2 (4.2)	0.828	1 (2.1)	0.857
No	35 (3.6)		17 (1.7)	
Renal failure (%)				
Yes	12 (6.4)	0.023	7 (3.7)	0.022
No	25 (3.0)		11 (1.3)	
Emergency (%)				
Yes	5 (3.1)	0.704	3 (1.9)	0.914
No	32 (3.7)		15 (1.7)	
Revision surgery (%)				
Yes	23 (29.5)	0.000	18 (23.1)	0.000
No	14 (1.5)		0 (0.0)	
Transfusions (%)				
≥ 5	14 (11.8)	0.000	10 (8.4)	0.000
< 5	23 (2.5)		8 (0.9)	
Duration of intubation (%)				
≥ 24 h	9 (5.7)	0.118	6 (3.8)	0.031
< 24 h	28 (3.2)		12 (1.4)	

The significant risk factors from the univariate analysis were included in a multiple logistic regression analysis (Table 4). Four risk factors could be confirmed as independent predictors for SSI CRP ≥ 1 mg/dL (p = 0.013), peripheral arterial disease (p = 0.039), operating time (p = 0.007) and revision operation (p = 0.000).

**Table 4:** Multiple logistic regression analysis of risk groups

	B	SD	Wald	p	OR	95%	CI
CRP	− 0.15	0.06	6.11	0.013	0.857	0.759	0.969
PAD	1.11	0.54	4.26	0.039	30.28	1.058	8.666
Myocardial infarction within the past half year	− 0.11	0.49	0.05	0.824	0.896	0.341	2.357
Renal failure	0.56	0.46	1.45	0.228	1.749	0.705	4.337
Incision/suture time	− 0.01	0.003	7.31	0.007	0.993	0.987	0.998
Revision surgery	3.42	0.45	56.66	0.000	30.536	12.537	74.377
Total of transfusions	− 0.01	0.04	0.16	0.690	0.986	0.919	1.058
Duration of intubation	0.01	0.01	1.77	0.183	1.007	0.997	1.016
Constant	− 3.77	1.69	4.98	0.026	0.023		

B = coefficient of the logistic regression, SD = standard deviation, Wald = Wald-test, p = p-value, OR = odds ratio, CI = confidence interval

Within the previously identified risk factors no statistically significant differences between the cefuroxime and cefazolin group regarding SSI in DSWI could be evaluated (Additional file 1: Tables S4). In all cases of SSI microbiological analyses were performed to identify the organisms responsible for the infections. Meticillin-resistant *Staphylococcus epidermidis* (MRSE) and Meticillin-sensitive *Staphylococcus aureus* (MSSA) were the leading microbial causes for DSWI (Additional file 1: Tables S5).

## Discussion

The right antibiotic choice for reducing the risk of DSWI in cardiac surgery has been subject to a large number of studies in the last decades. Since placebo-controlled trials have demonstrated a significant advantage the administration of antibiotics before incision has become an obligate standard. These studies mainly originated in the 1970s and prove ß-lactamase antibiotics to be the preferred class of antibiotics for this kind of indication [1, 2, 12]. While meta-analyses indicate that cephalosporins are superior to glycopeptides (e.g. vancomycin) when it comes to overall chest infections with no resistant organisms (e.g. MRSA or MRSE) [13], the question of the right generation cephalosporin remains unproven. Different generations of cephalosporins showed no apparent differences but the trend should go towards earlier-generation cephalosporins taking the most responsible organism *Staphylococcus aureus* into account [1, 2], provided the strains are Meticillin-sensitive. Page et al. suggested the use of cefazolin in standard cases [14]. Dellinger et al. as well as Mangram et al. consented to an overall recommendation of cefazolin for everyday use in standard cases [15, 16]. The latest study on the topic has been performed by Sommerstein et al. [17]. This prospective cohort study was able to include 14 centers all over Switzerland enrolling a total of 21.007 patients undergoing cardiac surgery. The study was overviewed and controlled by the ‘Swissnoso’ center for infection surveillance. Regarding the choice of the antimicrobial substance cefazolin was compared to cefuroxime and a combination of vancomycin and cefuroxime. The overall rates for SSI ranged between 3.4% to 3.8% whereas DSWI occurred in 1.3% to 2.1% of the cases. For all SSI cefazolin showed a significant reduction of infection rates, for DSWI cefazolin and the combination of vancomycin and cefuroxime achieved significantly better results than cefuroxime. In any case the second-generation cephalosporin had statistically significant higher infection rates. Not only from the antimicrobial stewardship perspective second-generation cephalosporins should only be used if a stronger gramnegative coverage is needed. As the use of antibiotics for surgical prophylaxis is mainly safe and clearly benefits the patient, the antibiotic selection pressure is not to be underestimated especially in the way of the well-known documented issue of prolonging antibiotic prophylaxis far beyond wound closure [18, 19]. In order to optimize the antibiotic consumption, one of the strategies initiated by our AMS was the implementation of hospital guidelines on the use of antibiotics for surgical prophylaxis and treatment of infections considering the local infection control surveillance and resistance data. The standardization of the PAP included not only the prophylactic agent but also recommendations about its postoperative restriction (depending on the type of surgery). The exception from the rule involved all cardiac procedures for their postoperative duration of less than 24 h retained as before the protocol change. The switch of the prophylactic drug was completely met with acceptance: we reached an adherence of 100%. Although there was no significant difference in the total rate of SSI, DSWI were less observed in the cefazolin group.

The most important limitation to this trial is the retrospective study design. The follow-up time included only the admission period. SSI developed post-discharge were only included and evaluated if the patients presented themselves and were treated in the same surgical center. Another disadvantage of this design is that diagnostic criteria have not been defined beforehand, including patient data. Furthermore some import data is missing as to details of ways of performing CABG (e.g. use of unilateral versus bilateral internal thoracic artery, approaches for bybass grafting or use of cardiopulmonary bybass versus off-pump procedures).

## Conclusions

In conclusion, the study reflects the roll-out of a hospital wide new antibiotic policy carried out by a recently enforced AMS with promising results for the most dreaded of postoperative complications in cardiac surgery when used cefazolin as PAP.

Furthermore a shorter regimen (one postoperative dose) may be effective enough, although the nature of this analysis demands for evident proof from additional studies.

## Supplementary Information

**Additional file 1:** Extended patient and microbiological dataset.

## Data Availability

The datasets used and/or analysed during the current study are available from the corresponding author on reasonable request. All data generated or analysed during this study are included in this published article [and its supplementary data files].

## Abbreviations

ASP: Antimicrobial stewardship program
DSWI: Deep sternal wound infections
PAP: Perioperative antimicrobial prophylaxis
SAP: Surgical antimicrobial prophylaxis
SSI: Surgical site infections
MSSA: Meticillin-sensitive *Staphylococcus aureus*
MRSA: Meticillin-resistant *Staphylococcus aureus*
MRSE: Meticillin-resistant *Staphylococcus epidermidis*

## Declarations

### Transparency declaration

None to declare.
