# Supplementary material for: Antimicrobial stewardship measures in cardiac surgery and its impact on surgical site infections
Source: J Cardiothorac Surg. 2021 Oct 20;16:309. doi: 10.1186/s13019-021-01693-7 (PMC8527641; doi:10.1186/s13019-021-01693-7)
Supplement: Supplementary file 1 — Additional file 1: Extended patient and microbiological dataset. [file 13019_2021_1693_MOESM1_ESM.docx]

Antimicrobial stewardship measures in cardiac surgery and its impact on surgical site infections

G. Surat^1^, D. Bernsen^2^, C. Schimmer^3^

1. Department of Infection Control and Antimicrobial Stewardship, University Hospital of Würzburg, Würzburg, Germany
2. Julius-Maximilians-University Würzburg, Würzburg, Germany
3. Department of Cardiothoracic and Thoracic Vascular Surgery, University Hospital of Würzburg, Würzburg, Germany

- Corresponding author. G. Surat. E-Mail address: [surat_g@ukw.de](mailto:surat_g@ukw.de)

**Supplementary Data**

|  | **Total** | **Cefuroxime** | **Cefazolin** | **p** |
| --- | --- | --- | --- | --- |
| **COPD (%)** | 94 (9.1) | 56 (9.6) | 38 (8,5) | 0.531 |
| **PAD (%)** | 96 (9.3) | 51 (8.8) | 45 (10.1) | 0.476 |
| **Diabetes mellitus (%)** | 324 (31.5) | 182 (31.3) | 142 (31.8) | 0.865 |
| **Steroid therapy (%)** | 10 (1.0) | 7 (1.2) | 3 (0.7) | 0.389 |
| **Nicotine abuse (%)** | 345 (33.5) | 178 (30.6) | 167 (37.4) | 0.022 |
| **Myocardial infarction within the past half year (%)** | 234 (22.7) | 129 (22.2) | 105 (23.5) | 0.615 |
| **Ejection fraction <30% (%)** | 48 (4.7) | 26 (4,5) | 22 (4.9) | 0.732 |
| **Cardiogenic shock (%)** | 20 (1.9) | 12 (2.1) | 8 (1.8) | 0.754 |
| **Renal failure (%)** | 188 (18.3) | 113 (19.4) | 75 (16.8) | 0.278 |

Table 1. Patient comorbidities

|  | **Total** | **Cefuroxime** | **Cefazolin** | **p** |
| --- | --- | --- | --- | --- |
| **Bypass surgery** | 637 (61.9) | 353 (60.7) | 284 (63.5) |  |
| **Procedure (%) Valve surgery** | 225 (21.9) | 143 (24.6) | 82 (18.3) | 0.038 |
| **Combination** | 167 (16.2) | 86 (14.8) | 81 (18.1) |  |
| **Emergency surgery (%)** | 162 (15.7) | 86 (14.8) | 76 (17.0) | 0.331 |
| **Incision/suture time (min)** | 226 ± 61 | 230 ± 64 | 223 ± 55 | 0.188 |
| **HLM time (min)** | 118 ± 46 | 118 ± 47 | 118 ± 45 | 0.844 |
| **X-clamp time (min)** | 82 ± 33 | 81 ± 32 | 83 ± 34 | 0.357 |

Table 2. Intraoperative patient data

|  | **Total** | **Cefuroxime** | **Cefazolin** | **p** |
| --- | --- | --- | --- | --- |
| **Revision surgery (%)** Bleeding | 59 (5.8) | 34 (5.9) | 25 (5.7) | 0.899 |
| CRC | 1.54 ± 2.83 | 1.52 ± 2.61 | 1.56 ± 3.11 | 0.076 |
| **Transfusions**  PC | 0.28 ± 0.86 | 0.24 ± 0.76 | 0.34 ± 0.99 | 0.141 |
| FFP | 0.10 ± 0.71 | 0.10 ± 0.71 | 0.10 ± 0.71 | 0.807 |
| **Duration of intubation (h)** | 23.95 ± 43.5 | 21.25 ± 37.1 | 27.50 ± 50.5 | 0.000 |
| **Heart drainage flow rate (mL)** | 810 ± 731 | 762 ± 667 | 875 ± 804 | 0.007 |
| **Postoperative delirium (%)** | 111 (10.8) | 75 (12.9) | 36 (8.1) | 0.013 |

Table 3. Postoperative patient data

| **Infections** |  | **SSI** | **p** | **DSWI** | **p** |
| --- | --- | --- | --- | --- | --- |
| **CRP ≥1mg/dL (%)** | Group 1 | 8 (8.3) | 0.570 | 5 (5.2) | 0.215 |
|  | Group 2 | 4 (6.0) |  | 1 (1.5) |  |
| **PAD (%)** | Group 1 | 4 (7.8) | 0.853 | 2 (3.9) | 0.633 |
|  | Group 2 | 4 (8.9) |  | 2 (2.2) |  |
| **Myocardial infarction** | Group 1 | 7 (5.4) | 0.819 | 6 (4.7) | 0.250 |
| **within the past half year (%)** | Group 2 | 5 (4.8) |  | 2 (1.9) |  |
| **Renal failure (%)** | Group 1 | 7 (6.2) | 0.897 | 4 (3.5) | 0.870 |
|  | Group 2 | 5 (6.7) |  | 3 (4.0) |  |
| **Incision/suture time** | Group 1 | 16 (7.2) | 0.991 | 9 (4.0) | 0.158 |
| **≥240min (%)** | Group 2 | 10 (7.1) |  | 2 (1.4) |  |
| **Revision surgery (%)** | Group 1 | 13 (27.1) | 0.556 | 12 (25.0) | 0.610 |
|  | Group 2 | 10 (33.3) |  | 6 (20.0) |  |
| **Total of transfusions ≥5** | Group 1 | 5 (7.9) | 0.169 | 4 (6.3) | 0.392 |
|  | Group 2 | 9 (16.1) |  | 6 (10.7) |  |
| **Duration of intubation ≥24h** | Group 1 | 2 (2.8) | 0.143 | 2 (2.8) | 0.530 |
|  | Group 2 | 7 (8.2) |  | 4 (4.7) |  |

Table 4. Primary endpoints in confirmed risk groups

| **Pathogen** | **Number of wound infections** |
| --- | --- |
| Meticillin-resistant *Staphylococcus epidermidis* | 6 |
| Meticillin-sensitive *Staphylococcus aureus* | 4 |
| *Propionbacterium acnes* | 3 |
| *Pseudomonas aeruginosa* | 2 |
| *Streptococcus viridans* | 1 |
| *Escherichia coli* | 1 |
| *Staphylococcus hominis* | 1 |
| *Serratia marcescens* | 1 |
| *Streptococcus anginosus* | 1 |
| No pathogen detected | 3 |

Table 5. Microbiological findings in DSWI
